# Genetic diversity of ‘Very Important Pharmacogenes’ in two South-Asian populations

**DOI:** 10.7717/peerj.12294

**Published:** 2021-11-10

**Authors:** Neeraj Bharti, Ruma Banerjee, Archana Achalere, Sunitha Manjari Kasibhatla, Rajendra Joshi

**Affiliations:** High Performance Computing: Medical & Bioinformatics Applications Group, Centre for Development of Advanced Computing, Pune, Maharashtra, India

**Keywords:** GIH, ITU, 1000 Genomes Project, gnomAD, SNPs, Allele frequency, Variant calling, Pharmacogenes

## Abstract

**Objectives:**

Reliable identification of population-specific variants is important for building the single nucleotide polymorphism (SNP) profile. In this study, genomic variation using allele frequency differences of pharmacologically important genes for Gujarati Indians in Houston (GIH) and Indian Telugu in the U.K. (ITU) from the 1000 Genomes Project vis-à-vis global population data was studied to understand its role in drug response.

**Methods:**

Joint genotyping approach was used to derive variants of GIH and ITU independently. SNPs of both these populations with significant allele frequency variation (minor allele frequency ≥ 0.05) with super-populations from the 1000 Genomes Project and gnomAD based on Chi-square distribution with *p-*value of ≤ 0.05 and Bonferroni’s multiple adjustment tests were identified. Population stratification and fixation index analysis was carried out to understand genetic differentiation. Functional annotation of variants was carried out using SnpEff, VEP and CADD score.

**Results:**

Population stratification of VIP genes revealed four clusters viz., single cluster of GIH and ITU, one cluster each of East Asian, European, African populations and Admixed American was found to be admixed. A total of 13 SNPs belonging to ten pharmacogenes were identified to have significant allele frequency variation in both GIH and ITU populations as compared to one or more super-populations. These SNPs belong to VKORC1 (rs17708472, rs2359612, rs8050894) involved in Vitamin K cycle, cytochrome P450 isoforms CYP2C9 (rs1057910), CYP2B6 (rs3211371), CYP2A2 (rs4646425) and CYP2A4 (rs4646440); ATP-binding cassette (ABC) transporter ABCB1 (rs12720067), DPYD1 (rs12119882, rs56160474) involved in pyrimidine metabolism, methyltransferase COMT (rs9332377) and transcriptional factor NR1I2 (rs6785049). SNPs rs1544410 (VDR), rs2725264 (ABCG2), rs5215 and rs5219 (KCNJ11) share high fixation index (≥ 0.5) with either EAS/AFR populations. Missense variants rs1057910 (CYP2C9), rs1801028 (DRD2) and rs1138272 (GSTP1), rs116855232 (NUDT15); intronic variants rs1131341 (NQO1) and rs115349832 (DPYD) are identified to be ‘deleterious’.

**Conclusions:**

Analysis of SNPs pertaining to pharmacogenes in GIH and ITU populations using population structure, fixation index and allele frequency variation provides a premise for understanding the role of genetic diversity in drug response in Asian Indians.

## Introduction

Pharmacogenomics approaches enable understanding the spectrum of genetic diversity responsible for drug response (Roden et al., 2011). The advent of high throughput sequencing technologies enabled population-scale sequencing which led to efforts like the 1000 Genomes Project (1000 Genomes Project Consortium et al., 2015) and gnomAD (Karczewski et al., 2020) that have in-turn provided opportunities to probe genetic diversity in previously understudied populations. Owing to such advances, precision public health is gaining acceptance and the focus is now shifting from disease treatment to its prevention and early detection (Khoury, Iademarco & Riley, 2016). Most of the clinical trials for drug responses are majorly conducted in populations of European descent (Thiers, Sinskey & Berndt, 2008). Even though the need for large-scale ‘megatrials’ across different populations has been understood, factors like lack of resources, insufficient expertise and under-powered studies have hindered the implementation of the same (Allison, 2012). Adverse drug response due to pharmaco-ethnic influence of population-specific variations has been well-documented for anticancer agents and warfarin (Kaye et al., 2017; Huang & Ratain, 2009). Clues towards predicting variable drug response due to influence of genetic structure of population(s) have been reported previously (Bachtiar et al., 2019; Wilson et al., 2001). The focus of precision public health is intervention at the population-level. Hence, understanding the genetic landscape of pharmacogenomic variants promises to tailor population-based pharmacogenomic interventions and testing (Nagar et al., 2019; Sivadas & Scaria, 2019).

In this context, the knowledge of genetic diversity amongst Indian sub-continent population and understanding its complex population structure are valuable as the sub-continent constitutes 20% of the world population (Sengupta et al., 2016; Banerjee, 2011; Majumder, 2010). There are few genome wide association studies (GWAS) carried out for understanding the role of allele variation in populations pertaining to Indian subcontinent (Prasad et al., 2019; Nagrani et al., 2017; Giri et al., 2016). Such studies aid in hypothesis-free detection of genetic variant catalog and provide insight into pleiotropy. It needs to be mentioned that the resolution of causal variants derived using GWAS is influenced by cohort constitution, secondary diseases, environmental variations along with ethnic diversity (Wijmenga & Zhernakova, 2018; Gamazon & Perera, 2012).

The 1000 Genomes Project (1KGP) provides data of 26 ethnic groups spread across the globe with an aim to capture genetic variants with frequencies of at least 1% in the population (1000 Genomes Project Consortium et al., 2015). Similarly, resources like gnomAD include aggregated and harmonized datasets of both disease-specific as well as large-scale population genomics studies (Karczewski et al., 2020). Samples included in 1KGP have varied coverage ranging from low (2-4X) to high (50X). Joint variant calling overcomes challenges associated with low-coverage by providing a consistent set of calls at all possible sites (Chen, Boehnke & Fuchsberger, 2020). In 1KGP samples were selected from different ethnic groups and annotated as ‘population’. These ‘populations’ were then grouped together on the basis of geographical location into ‘super-population’ and allele frequencies reported in 1KGP are derived based on super-population information (1000 Genomes Project Consortium et al., 2015). The ∼85 million variants listed in Phase 3 of 1KGP were obtained by taking into consideration ∼2500 samples belonging to all the ethnic groups included in the study (derived based on five super-populations). The ‘super-population derived variant set’ may have lower resolution to ascertain individual population-specific variants that may be responsible for adaptation to the local environment. Hence, variant profiles obtained after joint variant detection of ‘individual populations’ promise to provide a more precise call set of variants for population genomics studies based on comparison of allele frequencies.

Allele frequency variation is a complementary measure to conventional metrics like fixation index (*Fst*) and is proposed to be a robust population differentiation parameter (Berner, 2019). Fixation index hints at the proportion of total genetic variation at a given locus between populations and is influenced by minor allele frequency (MAF) and population sample size (Berner, 2019). Population stratification approaches are known to provide a framework to understand genetic differentiation based on admixture patterns by taking into account complex evolutionary models (Grünwald et al., 2017). Hence a combined approach of allele frequency comparison, fixation index calculation and population structure has been used in this study.

The present work is an effort towards cataloguing genetic variants and their distribution across two ethnic groups of Indian ancestry *i.e.*, Gujarati Indian from Houston, Texas (GIH) and Indian Telugu in the U.K. (ITU) as compared to the combined data set of global variants. GIH population was chosen as it occupies a unique position in the genetic ancestry of Indian subcontinent due to its preponderance of ancient North Indian gene pool as compared to the rest of the subcontinent (ITU) which has ancient South Indian ancestry (Silva et al., 2017; Reich et al., 2009). There are reports of underestimation of genetic diversity of Indian sub-continent in 1KGP owing to the fact that GIH and ITU along with Sri Lankan Tamil in the UK (STU) have been sampled from Indian diaspora wherein a major driver of social hierarchy in India *i.e.*, caste/tribe and endogamy are not observed (Sengupta et al., 2016). However in the absence of availability of more appropriate samples in the public domain we have used 1KGP data. GIH and ITU are part of South Asian (SAS) super-population which also includes Punjabis in Lahore (PJL), Bengali in Bangladesh (BEB) and STU populations. Allele frequencies of GIH and ITU in 1KGP are hence influenced by cohort constitution of other populations in the SAS group. As we are interested in ascertaining individual population-specific variants of GIH and ITU, independent joint variant calling of GIH and ITU was performed.

Our group has earlier analysed skin pigmentation related genes for positive selection in GIH and ITU populations (Jonnalagadda et al., 2017). In the present study, we attempt to prioritize single nucleotide polymorphism (SNPs) associated with very important pharmacogenes (VIP) in terms of allele frequency variation between populations and fixation index. The study of such variants in the GIH and ITU populations would help to deduce the underlying pattern/distribution and aid in understanding the landscape of genetic variation in pharmacologically important genes in Indian subcontinent.

## Materials and Methods

### Variant calling

**Figure 1:**
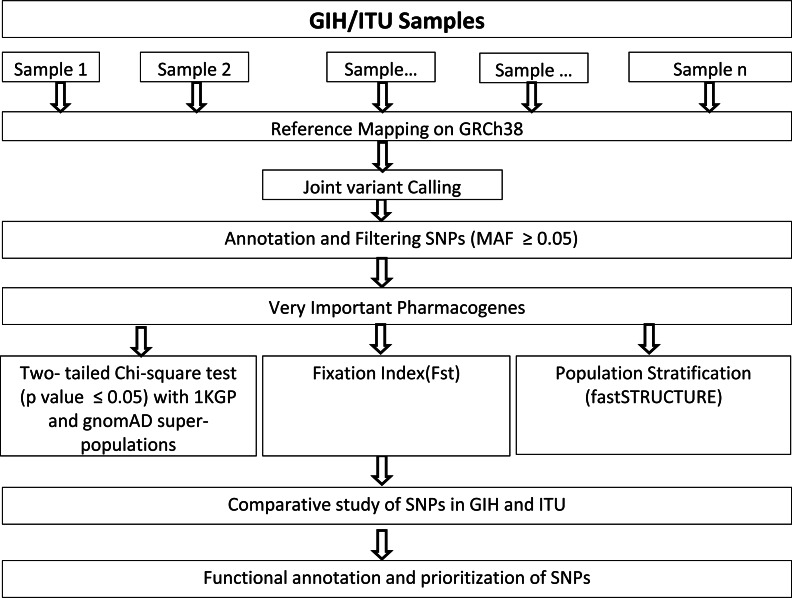
Flow-chart for identification and analysis of SNPs pertaining to VIP genes.

Genome alignments of 109 and 112 samples belonging to South Asian descent namely GIH and ITU respectively included in the Phase 3 of 1KGP (available as of December 2018) were used for joint variant calling with human genome build GRCh38 as reference. Only autosomal chromosomes were included in this study. It should be mentioned that low-coverage samples were included in this study as high-coverage data was available only for a small proportion of the samples. Joint variant calling of low-coverage samples was carried out using GATK-3.8 (McKenna et al., 2010). GATK-HaplotypeCaller was run per sample resulting in the generation of an intermediate output in genomic variant calling format (GVCF). HaplotypeCaller was used with default parameters for depth and mapping quality. Joint genotyping was carried out independently for GIH and ITU populations using GenotypeGVCFs (with default parameters) using individual sample GVCF files as input (Fig. 1). It must be mentioned that only SNPs were used for further analysis and indels were excluded in this study.

### Variant filtering and annotation

Variants were filtered based on minimum allele frequency (MAF) ≥ 0.05 in GIH and ITU populations. To understand the variation in the SNP profile across samples, principal component analysis (PCA) was performed using R package snprelate (Zheng et al., 2012). Phasing of variants was carried out using Beagle 5.0 (Browning, Zhou & Browning, 2018). Annotation was carried out using SnpEff 4.3 (Cingolani et al., 2012) and VEP (McLaren et al., 2016) along with dbSNP build 154 (Sherry et al., 2001). The variants were sorted according to chromosome number along with genic and intergenic regions that were obtained using SnpSift (Cingolani et al., 2012). Variants were annotated with CADD scores using GRCh38-v1.6 database and variants with score ≥ 15 were considered deleterious (Rentzsch et al., 2021).

### Gene-set

Genes categorised as ‘Very Important Pharmacogenes’ listed in PharmGKB (Whirl-Carrillo et al., 2012) have been retrieved. This data was further filtered to remove genes part of chromosome X and mitochondria which resulted in 65 genes.

### Populations analysed

1KGP: Samples pertaining to European (EUR), East Asian (EAS), African (AFR), Ad Mixed American (AMR) were analysed. Ad Mixed Americans were further divided into two “subpopulations” based on ancestry viz., European-derived (CLM and PUR) and Latino (MXL and PEL) respectively as these are known to be genetically different (Gómez et al., 2021) (File S1A).

gnomAD v3: Samples pertaining to European (EUR), East Asian (EAS), African (AFR), Ad Mixed American (AMR), Amish (AMI), Ashkenazi Jewish (ASJ), European-Finnish (FIN), European-non-Finnish (NFE), Other (OTH) and South Asian (SAS) were analysed. (File SB) gnomAD v3 was included for comparison of allele frequency variation across different populations as this database is a more extensive resource when compared to 1KGP (which has ∼2500 samples as of December 2018 that are part of 5 super-populations). gnomAD v3 in comparison has 76,156 samples pertaining to 9 populations (available as of September 2020). Hence in order to take into account existing samples available in the public domain databases we included gnomAD for comparison of allele frequency variation of VIP genes in our study.

### Test of significance, prioritization and functional annotation

For the comparison of allele frequencies, variants listed in the Phase 3 of 1KGP database (derived from 26 populations) and gnomAD (v3) were used as reference sets. The current version of gnomAD includes only genome samples with >18X coverage (and hence do not include 1KGP genome data). It is to be noted that the earlier version of this database (v2.1.1) has a wider coverage but was not included in this study as GRCh37 was used as the reference genome. Allele frequency variation was calculated only for SNPs annotated in PharmGKB (URL: https://api.pharmgkb.org/v1/download/file/data/variantAnnotations.zip). The difference in allele frequencies of the GIH and ITU populations with respect to other super-populations in 1KGP and gnomAD were calculated in terms of Chi-square statistics. To capture significant allele frequency differences between the GIH/ITU and other super-populations, two-way Chi-square values were calculated wherein GIH population allele frequencies and “super-population derived allele frequencies” were compared with respect to each other as observed and expected values. Thus Chi-square statistics of variants were obtained by cumulative *χ*^2^ values for both the scenarios of observed frequencies of GIH/ITU population(s) alleles and super population alleles. Then under the null hypothesis of Chi-square distribution, *p*-values associated with *χ*^2^ statistics of all the variants were calculated. Statistically significant variants were obtained for all those Chi-square distributions of individual populations using *p*-value of ≤ 0.05.

The SNPs with *p*-value ≤ 0.05 were corrected using Bonferroni’s multiple tests to calculate the level of significance (*p* ≤ (0.05/(#variants ×#super-populations)). Alleles with frequency in the range of 5–100% (Sachidanandam et al., 2001) in GIH and ITU populations that satisfied the *p*-value cut-off of ≤ 0.05 were analysed further. SNPs absent in populations other than GIH and ITU were assigned allele frequency values of 10^−10^ in order to enable calculation of Chi-square statistics. Comparative analysis of significant SNPs in GIH and ITU were carried out and SNPs unique as well as shared between GIH and ITU populations were analysed further based on their annotation. Significant SNPs were also mapped with ClinVar database (Landrum et al., 2014) to obtain clinical association, if any.

### Population stratification

fastSTRUCTURE which is based on variational Bayesian framework was used to infer the population structure of the VIP genes (Raj, Stephens & Pritchard, 2014). PGDSpider (2.1.1.5) was used for input file preparation for fastSTRUCTURE (Lischer & Excoffier, 2012). Simple prior was used with (*k*) 1 to 10. Optimal values of *k* were selected based on maximum likelihood values and membership coefficient values ≥ 0.05 were assessed. Genetic differentiation was analysed using fixation index which was calculated using VCFtools (v0.1.16) (Danecek et al., 2011), that implements Weir and Cockerham’s unbiased estimator (Cadzow et al., 2014; Weir & Cockerham, 1984).

## Results

### Joint genotyping

Genome-wide joint variant calling of GIH and ITU populations independently predicted 7,319,189 and 7,228,257 SNPs in GIH and ITU respectively with MAF ≥ 0.05. Comparison of these variants with that listed in Phase 3 data of 1KGP revealed 5,602,124 and 5,638,042 SNPs to be common with variants predicted using joint genotyping of GIH and ITU respectively. Similar observation was noted during comparison of joint genotyping of GIH and ITU variants with gnomAD, wherein 6,59,4122 and 6,64,8248 SNPs respectively were found to be common. Of these 12286 (GIH) and 12144 (ITU) belong to VIP genes. The variant set was filtered further based on variants listed in PharmGKB which resulted in 250 and 249 SNPs in GIH and ITU respectively (Table 1). This variant dataset was used for analysing population structure and for comparison of allele frequency variation across super-populations to understand SNPs with significant allele frequency variation and fixation index.

**Table 1:** SNPs in GIH and ITU at each filtering step for both genome-wide and VIP datasets.

**SNP filtering step**	**#SNPs in GIH**	**#SNPs in ITU**
**Genome-wide SNPs**		
MAF ≥0.05	7,319,189	7,228,257
Common with 1KGP	5,602,124	5,638,042
Common with gnomAD	6,59,4122	6,64,8248
**Very Important Pharmacogenes**		
MAF ≥0.05	12,286	12,144
Common with 1KGP	11,407	11,527
Common with gnomAD	12,050	12,179
Common with 1KGP and PharmGKB	250	249
Common with gnomAD and PharmGKB	262	261

**Figure 2:**
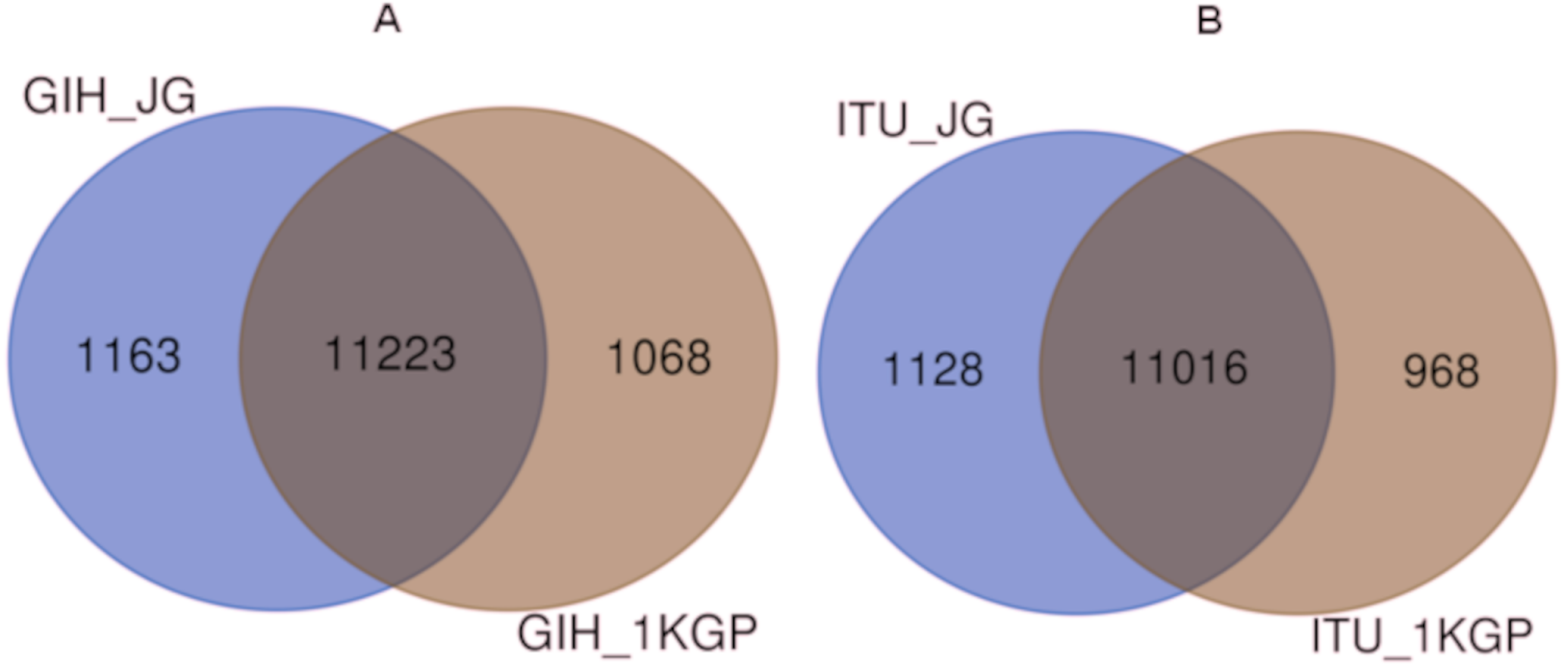
(A) Venn diagram depicting the common and unique SNPs belonging to VIP genes identified by joint genotyping of GIH population and that from 1KGP. (B) Venn diagram depicting the common and unique SNPs belonging to VIP genes identified by joint genotyping.

Comparison of variants of VIP genes obtained in this study (using population-specific genotyping of GIH and ITU) with corresponding samples in 1KGP (where in GIH and ITU are included in SAS super-population) revealed that for GIH and ITU 8–9% SNPs are unique in both the datasets (Fig. 2 and Files S2–S3). Missense variants rs2279343 (*CYP2B6*) and rs1801030 (*SULT1A1* involved in sulfate conjugation) are part of the exclusive SNPs identified by joint genotyping of GIH and ITU which are also annotated in PharmGKB variant list but absent in 1KGP (Files S2–S3).

### Population structure

**Figure 3:**
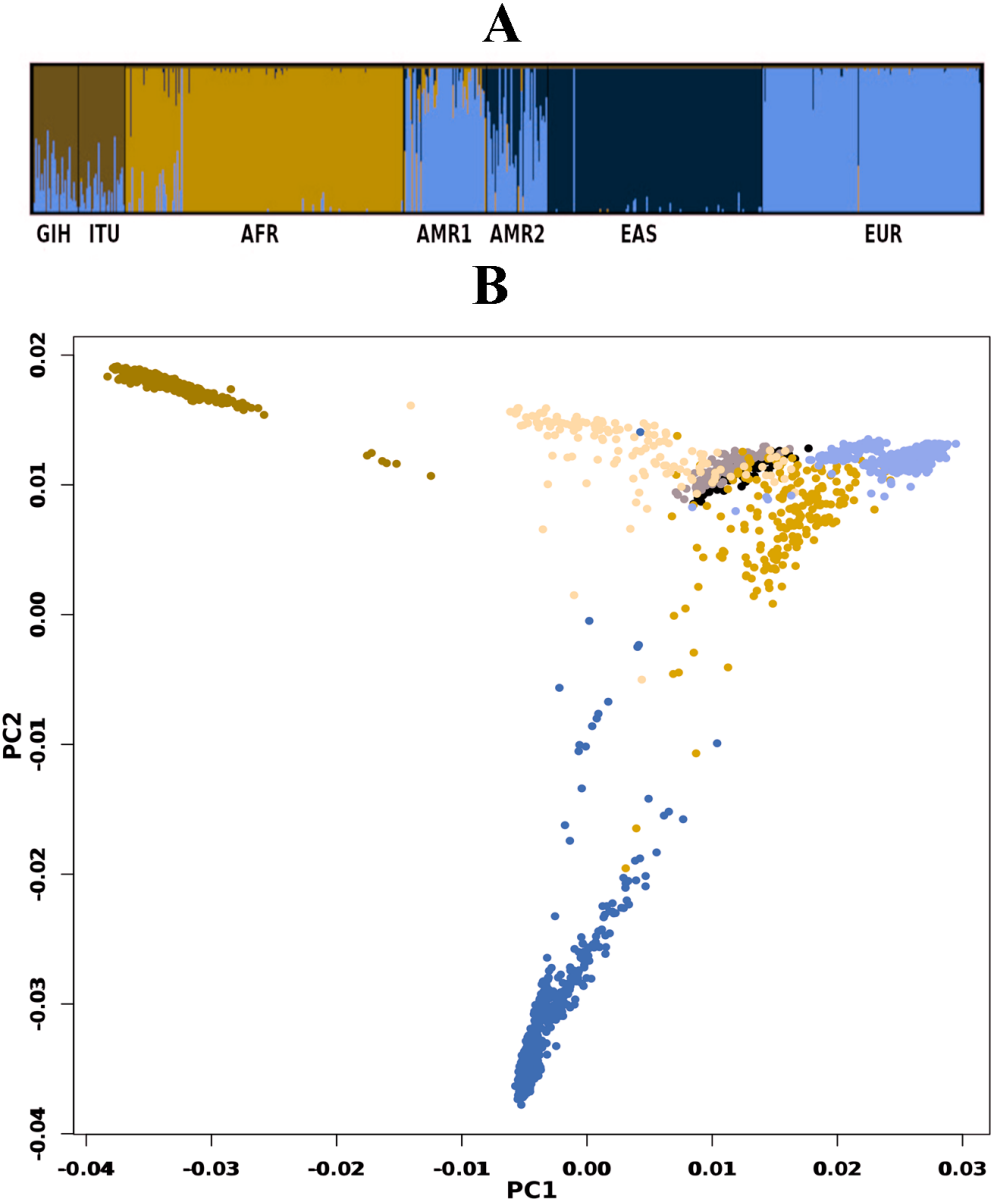
Genetic diversity of VIP genes. (A) Population stratification of VIP genes at *k* = 4; (B) Principal Component analysis of VIP genes. Color legend: AFR in dark blue, EUR in lavender, EAS in olive green, AMR1 in mustard, AMR2 in beige, GIH in black and ITU in stone grey.

A total of 163722 SNPs belonging to 65 VIP genes were used for population structure analysis. VIP variants were found to have stratified into *k* = 3 to 6 clusters (Fig. 3A, File S4). Optimal *k* = 4 was chosen based on the maximum number of individuals in a population having membership to a given cluster and marginal likelihood values. This resulted in the majority of GIH and ITU individuals being part of a single cluster (with few members reported to be admixed with EUR) (Fig. 3A). AMR1 and AMR2 are admixed with membership to two or more clusters (AFR, EUR and EAS). EUR, AFR and EAS are part of distinct clusters.

PCA of genome-wide SNPs revealed three major clusters viz., one each of AFR and EAS; the third cluster includes AMR1, AMR2, GIH, ITU and EUR (File S5). The first PC separates EAS from the rest of the populations whereas the second PC further separates AFR from other populations. When SNPs (#163722) pertaining to VIP genes were clustered using PCA, four clusters were observed that include three independent clusters of AFR, EUR and GIH/ITU. The fourth cluster consists of AMR1, AMR2 and EAS members (Fig. 3B).

### Allele frequency variation analysis across populations

**Figure 4:**
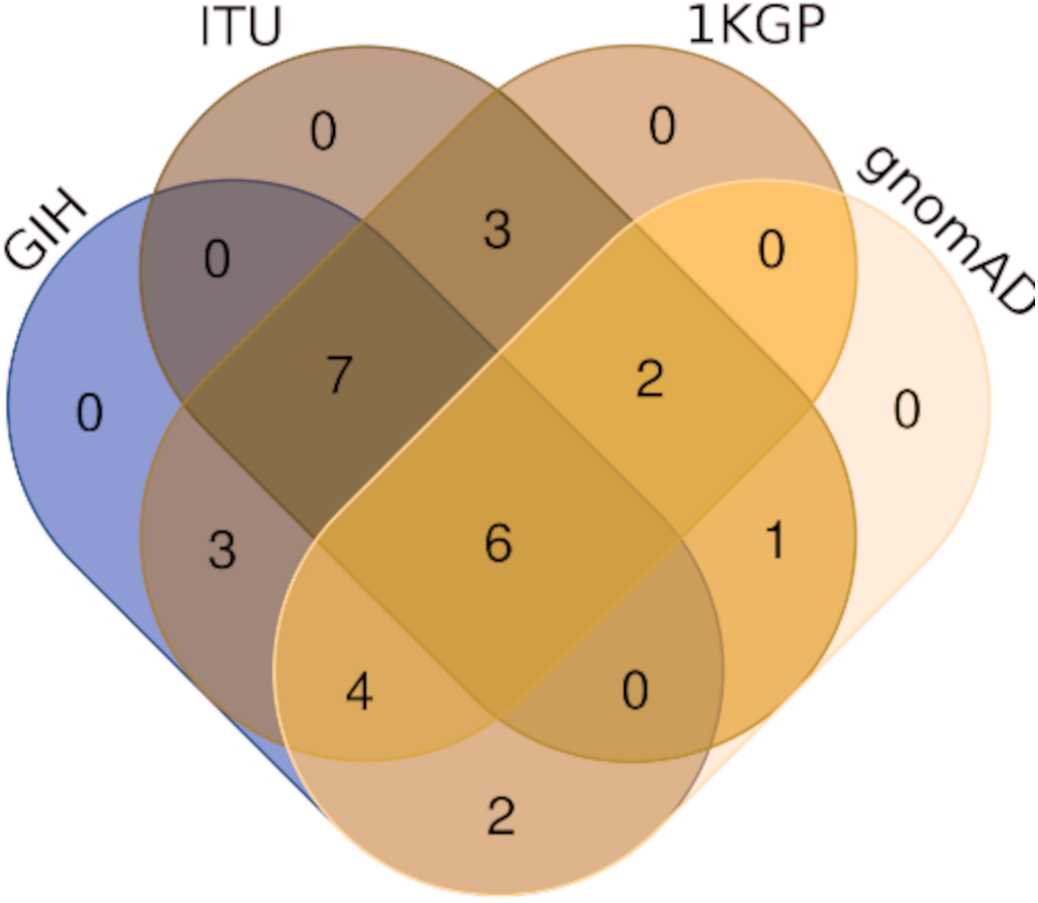
Venn diagram depicting SNPs with significant allele frequency variation in GIH and ITU with other populations/super-populations in 1KGP and gnomAD.

**Figure 5:**
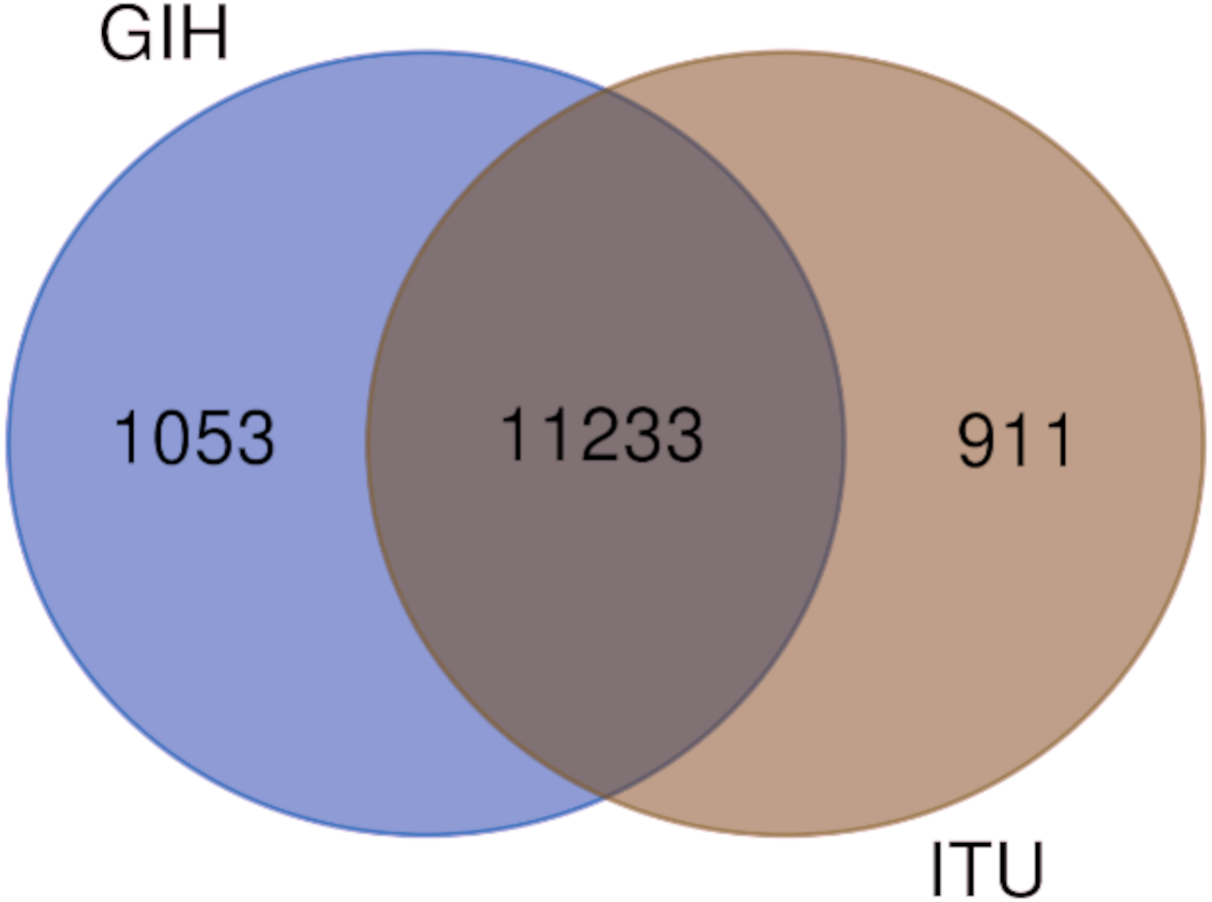
Venn diagram depicting shared and unique SNPs in GIH and ITU.

Of the 65 VIP genes analyzed in this study, 12286 and 12144 SNPs in GIH and ITU populations respectively have been obtained with MAF ≥ 0.05. Comparison of these SNPs with 1KGP and gnomAD populations/super-populations was carried out to identify shared and unique SNPs (Table 2, Fig. 4, File S6). The study revealed that ∼9% (#1053) SNPs are unique in the GIH population for the above mentioned gene set. Similarly, ∼8% (#911) SNPs are unique to the ITU population for the pharmacologically important genes (Fig. 5).

The proportion of MAFs of the variants for pharmacogenes is found to be higher as observed in other populations (Gravel et al., 2011). Moreover, the major allele frequency distribution amongst the populations remains comparatively undifferentiated. The number of SNPs with significant allele frequency variation (in GIH and ITU) is highest in AFR followed by EAS whereas AMR and EUR super-populations have comparatively lower numbers of SNPs. The trend of high differentiation of GIH and ITU with AFR and EAS super-populations agrees with ethnic, linguistic and similar factors (Ayub & Tyler-Smith, 2009).

### SNPs with significant allele frequency variation

#### SNPs with lower allele frequency in GIH and ITU

A total of seven SNPs with MAF ≤ 0.05 in GIH and ITU populations were found to have significant allele frequency variation in other populations or super-populations of 1KGP and gnomAD. In addition, seven and three SNPs in GIH and ITU are exclusively significant with one or more super-populations (File S7).

#### SNPs with higher allele frequency in both GIH and ITU

**Figure 6:**
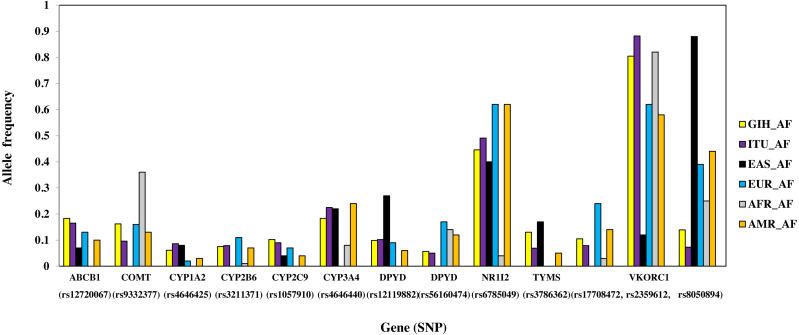
Histogram of SNPs (shared by GIH and ITU) belonging to VIP genes that show significant allele frequency variation with at least one super-population from 1KGP.

A total of 13 SNPs belonging to 10 genes have significant allele frequency variation in both GIH and ITU populations as compared to one or more super-populations (Table 2, Fig. 6). Majority of the shared SNPs are intronic except for one synonymous and two missense variants. These SNPs belong to *VKORC1* involved in Vitamin K cycle, cytochrome P450 isoforms *CYP2C9*, *CYP2B6*, *CYP2A1* and *CYP2A4*; ATP-binding cassette (*ABC*) transporter *ABCB1*, *DPYD1* involved in pyrimidine metabolism and transcriptional factor *NR1I2*. It is interesting to note that the CADD score for *CYP2C9* missense variant (rs1057910) is ∼17 and hence is identified as ‘deleterious substitution’.

**Table 2:** List of SNPs with significant allele frequency variation in GIH and ITU populations.

**ID**	**Annotation**	**Gene**	**CADD**	**REF**	**ALT**	**1KGP**	**gnomAD**
Significant SNPs in both GIH and ITU (# Only significant with GIH; *Only significant with ITU )							
rs1057910	Missense	CYP2C9	17.39	A	C	AFR	–
rs12119882	Intronic	DPYD	4.724	A	G	AFR	–
rs12720067	Intronic	ABCB1	0.45	C	T	AFR	–
rs17708472	Intronic	VKORC1	9.163	G	A	EAS	EAS#
rs2359612	Intronic	VKORC1	0.526	A	G	EAS	EAS
rs3211371	Missense	CYP2B6	0.341	C	T	EAS	–
rs3786362	Synonymous	TYMS	7.958	A	G	EAS and AFR	AMI, ASJ, FIN, NFE and AFR#
rs4646425	Intronic	CYP1A2	4.632	C	T	AFR	–
rs4646440	Intronic	CYP3A4	3.246	G	A	EUR	AMI, FIN, NFE and ASJ*
rs56160474	3′UTR	DPYD	2.272	A	G	EAS	–
rs6785049	Intronic	NR1I2	0.004	G	A	AFR	–
rs8050894	Intronic	VKORC1	0.72	C	G	EAS	EAS
rs9332377	Intronic	COMT	4.427	C	T	EAS	–
*Significant SNPs unique in GIH*							
rs1131341	Intronic	NQO1	23.7	G	A	AFR	–
rs1138272	Missense	GSTP1	19.3	C	T	EAS	EAS
rs115349832	Intronic	DPYD	17.92	A	C	EAS and AFR	AMR
rs1801028	Missense	DRD2	25.6	G	C	AFR	–
rs2231135	5′UTR	ABCG2	9.399	A	G	EAS	–
rs2884737	Intronic	VKORC1	1.955	A	C	EAS	–
rs9934438	Intronic	VKORC1	14.2	G	A	EAS	EAS
rs1709083	3′UTR	CYP2A13	0.73	C	G	–	AMR
rs2291075	Synonymous	SLCO1B1	7.098	C	T	–	AMI
*Significant SNPs unique in ITU*							
rs116855232	Missense	NUDT15	21.9	C	T	EUR and AFR	AMI
rs2293347	Synonymous	EGFR	9.124	C	T	AFR	–
rs2725264	Intronic	ABCG2	5.246	C	T	AFR	–
rs6018	Missense	F5	14.24	T	G	AFR	–
rs7294	3′UTR	VKORC1	1.521	C	T	EAS	EAS
rs1544410	Intronic	VDR	2	C	T	–	EAS

**Notes.**

EAS East Asian AFR African AMR Ad Mixed American AMI Amish ASJ Ashkenazi Jewish FIN European-Finnish NFE European-non-Finnish

#### SNPs with higher allele frequency in GIH

**Figure 7:**
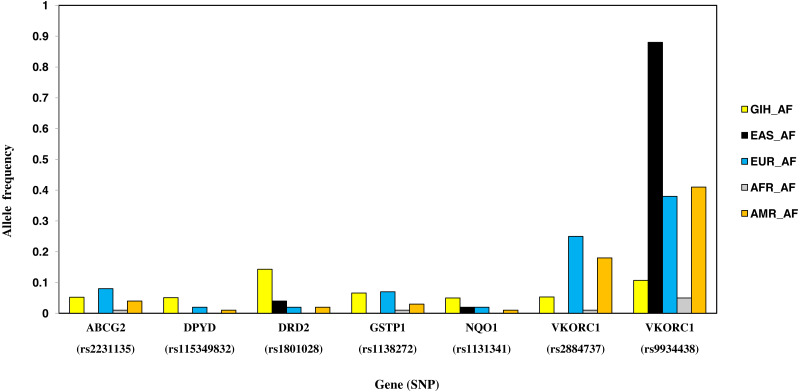
Histogram of SNPs in GIH population belonging to VIP genes that show significant allele frequency variation with at least one super-population from 1KGP.

Nine SNPs, part of eight genes, are unique to GIH with significant allele frequency variation when compared to one or more super-populations (Table 2, Fig. 7). Unique SNPs in GIH include two intronic SNPs of *VKORC1* and one intronic SNP of *DPYD*, two missense SNPs one each belonging to *GSTP1* and *DRD2*, one synonymous SNP of solute carrier *SLCO1B1*, 3′ UTR SNP in *CYP2A13* and 5′UTR SNP in *ABCG2*. Of these, missense variants rs1801028 (*DRD2*) and rs1138272 (*GSTP1*); intronic variants rs1131341 (*NQO1*) and rs115349832 (*DPYD*) have CADD score >15 and hence are predicted to be ‘deleterious’. Intronic SNP rs115349832 (*DYPD*) has been exclusively identified in the variant call-set obtained using joint genotyping of GIH (MAF ≥ 0.05). It is interesting to note that MAF of the same allele in 1KGP (for GIH) is 0.047 and would have been filtered as it does not satisfy the criteria of MAF ≤ 0.05 (File S2).

#### SNPs with higher allele frequency in ITU

**Figure 8:**
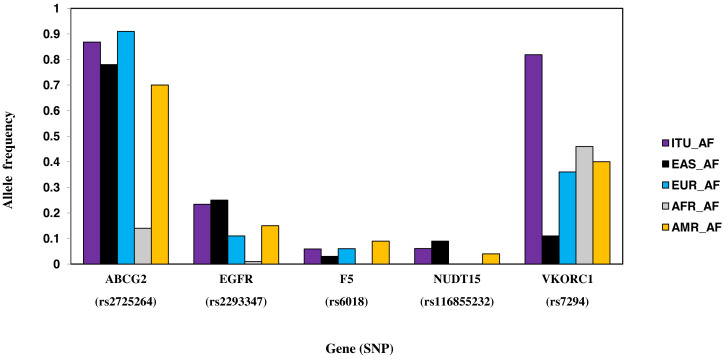
Histogram of SNPs in ITU population belonging to VIP genes that show significant allele frequency variation with at least one super-population from 1KGP.

Six SNPs, part of six genes, are unique to ITU (Table 2, Fig. 8). These include two missense SNPs one each in *NUDT15* and F5, two intronic SNPs one each in *ABCG2* and *VDR*, one synonymous SNP in *EGFR* and 3′ UTR SNP in *VKORC1*. Of these, the missense variant rs116855232 (*NUDT15*) is identified as deleterious (CADD score ≥15).

#### SNPs with fixation index ≥ 0.5

A total of 367 variants belonging to 39 genes with fixation index ≥ 0.5 in both GIH and ITU when compared with one or more super-populations were observed (Table 2, File S8). Of these ∼78% are intronic and ∼16% are intragenic SNPs whereas the rest include missense, synonymous and 3′/5′UTR SNPs. Seven SNPs viz., missense variant rs5219 (*KCNJ11*), intronic variants: rs74105153 (*DPYD*); rs2302535 (*EGFR*); rs12471933 and rs12466048 (*ALK*), 5′UTR variant rs75147926 (*BCR*) and 3′UTR variant rs712 (*KRAS*) are predicted to be ‘deleterious’ (CADD score ≥15). Genes *ALK*, *CFTR, EGFR, VDR, CYP2C9, ABCG2, DPYD* and *BCR* harbour more than 15 SNPs each with high fixation index (≥ 0.5).

## Discussion

Indian-subcontinent is one of the understudied regions in terms of exploring genetic diversity of native populations even though it constitutes a major proportion of the global population (Sengupta et al., 2016; Banerjee, 2011; Majumder, 2010). Understanding the spectrum of genetic variations in pharmacogenes is crucial for drug response studies (Wright et al., 2018). Population-level pharmacogenomics studies for understanding the dosage as well as drug adverse effects can be enabled by precision public health initiatives. In this study variants pertaining to ‘Very Important Pharmacogenes’ were computed for GIH and ITU populations from 1KGP and analysed based on allele frequency variation, fixation index and population structure with respect to other super-populations. Inclusion of a larger number of samples from gnomAD for comparison of allele frequency (derived using smaller dataset viz., 1KGP) provided a stronger measure of support for the observed variations.

**Table 3:** List of SNPs with Fst and significant allele frequency variation.

**Gene**	**ID**	**Annotation**	**CADD**	**GIH- AFR**	**GIH- AMR**	**GIH- EAS**	**GIH -EUR**	**ITU- AFR**	**ITU- AMR**	**ITU- EAS**	**ITU- EUR**	**GIH-ITU**
VDR	rs1544410	Intronic	2	0.04	0.05	0.38	0.00	0.13	0.14	**0.52**	0.02	0.02
ABCG2	rs2725264	Intronic	5.246	**0.59**	0.01	0.00	0.10	**0.68**	0.06	0.02	0.01	0.02
NR1I2	rs6785049	Intronic	0.004	**0.58**	0.05	0.00	0.05	**0.61**	0.03	0.01	0.04	0
VKORC1	rs2359612	Intronic	0.526	0.00	0.10	**0.68**	0.07	0.00	0.15	**0.72**	0.11	0
VKORC1	rs7294	3′UTR	1.521	0.09	0.14	**0.57**	0.18	0.21	0.28	**0.69**	0.31	0.04
VKORC1	rs8050894	Intronic	0.72	0.03	0.16	**0.72**	0.12	0.09	0.25	**0.77**	0.20	0.02
VKORC1	rs9934438	Intronic	14.2	0.02	0.19	**0.75**	0.16	0.00	0.25	**0.79**	0.21	0.01

**Notes.**

Fst values ≥ 0.5 are in bold.

EUR European EAS East Asian AFR African AMR Ad Mixed American GIH Gujaratis in Houston ITU Indian Telugu in the U.K

GIH and ITU were chosen to represent the North-Indian and South-Indian ancestry of the Indian sub-continent respectively (Reich et al., 2009). Overall, we observe that GIH and ITU group together as a homogenous population both in population stratification and in clustering using PCA (Fig. 3). Of the total VIP variants, only 8% in GIH and 7% in ITU have been found to be unique with MAF ≥ 0.05. The low proportion of distinct alleles in GIH and ITU can be attributed to samples being sourced from Indian diaspora which lack social hierarchy and endogamy, a prevalent factor in Indian sub-continent (Sengupta et al., 2016). Of the four clusters observed in population stratification, majority of the members of GIH and ITU formed a distinct cluster with a few members found to be admixed with EUR (Fig. 3A). GIH and ITU share similar allele frequencies for VIP genes with AMR1, AMR2 and EUR and hence significant allele frequency variation was observed predominantly with AFR and EAS. It is interesting to note that AFR and EAS remain as independent clusters even at larger *k* values (File S4). Majority of the SNPs (MAF ≥ 0.05) with significant allele frequency variation observed in our study had high values in GIH/ITU as compared to populations/super-populations of 1KGP and gnomAD. The converse scenario was only observed in SNPs with MAF ≤ 0.05 in GIH/ITU (File S7). This observation may vary when larger numbers of samples are taken into consideration for deriving allele frequencies.

Joint genotyping of individual populations (GIH and ITU independently) enabled identification of SNPs (with MAF ≥ 0.05) which hitherto would have been either filtered due to low allele frequency in 1KGP (rs115349832 of *DYPD*) or not identified in the dataset at all as observed in the case of rs2279343 (*CYP2B6*) and rs1801030 (*SULT1A1*) (Files S2–S3).

SNPs with significant allele frequency variation with EAS and high fixation index identified in this study include three intronic SNPs ( rs2359612, rs8050894, rs9934438) and one in 3′UTR ( rs7294) of *VKORC1* gene. Missense SNP rs1057910 (*CYP2C9*) along with the observed *VKORC1* variants have been associated with varied warfarin dosage in both South-Indian and North-Indian populations (Nizamuddin et al., 2021; Arun Kumar et al., 2015; Krishna Kumar et al., 2014; Giri et al., 2014; Shalia et al., 2012). Similarly, intronic SNP (rs6785049) present in *NR1I2* has significant allele frequency variation in AFR. *NR1I2* is a member of the nuclear receptor superfamily of transcriptional factors that regulates many genes like *CYP3A4*, a promiscuous cytochrome P450 enzyme involved in the metabolism of >50% drugs (Bertilsson et al., 1998). The AG genotype has a higher allele frequency in GIH and ITU. This genotype was found to be associated in patients with bladder cancer to have decreased exposure to temsirolimus or sirolimus as compared to patients with the GG genotype, and decreased likelihood of bone marrow and gastrointestinal toxicities, or other adverse events as compared to patients with the AA genotypes (Mbatchi et al., 2017). Also AG genotype has been found to be associated with increased risk for hypertension when treated with sunitinib as compared to patients with the GG genotype (Narjoz et al., 2015). It needs to be mentioned that so far there are no reports of association of this SNP with any phenotype in case of Indian population and hence this SNP is a good candidate to be probed for further validation studies.

SNP rs1544410 (Bsml) present in the intronic region of gene *VDR* has ≥ 0.5 fixation index with EAS in case of ITU population. Association of *VDR* polymorphism with diseases like tuberculosis, osteoporosis and obesity has been reported earlier (Uitterlinden et al., 2004). CT genotype has higher allele frequency in ITU population and this genotype is known to be associated with decreased response to drug deferasirox leading to higher liver stiffness in thalassemia major patients (Allegra et al., 2019). This genotype is also associated with increased likelihood of resistance when treated with clodronate in people with Osteitis Deformans (Mossetti et al., 2008). Ezhilarasi, Dhamodharan & Vijay (2018) have found Bsml to be associated with decreased levels of vitamin D circulation in Type 2 diabetic patients of South Indians. Gulati et al. (2020) have found Bsml to be associated with weight loss after lifestyle interventions in Asian Indians. Similarly, SNP rs2725264 present in the intronic region of gene *ABCG2* has ≥ 0.5 fixation index with AFR. *ABCG2* is an efflux protein involved in drug resistance to cancer treatment using platinum based drugs (Stacy, Jansson & Richardson, 2013). The pharmacokinetic effect of rs2231135 (5′UTR of *ABCG2*) in Asian cancer patients was found to have no major impact for three-week regimen of irinotecan (Jada et al., 2007). Role of this SNP in Indian population needs to be explored further taking into account the underlying ethnic diversity and social hierarchy.

Missense SNPs (rs5215 and rs5219) belonging to gene *KCNJ11* have ≥ 0.5 fixation index with AFR. Both these variants are found to be associated with Type 2 diabetes in EAS (Yang et al., 2012). However, Phani et al. (2014) did not find significant association in the South Indian population. It is interesting to note that rs5219 is predicted to be ‘deleterious’ and needs to be validated further to understand its role.

This study hence provides a catalog of significant variants in GIH and ITU populations for ‘Very Important Pharmacogenes’ that have a potential role in understanding the drug response in Indian populations. Further experimental studies of the variants need to be carried out to validate the findings. As allele frequencies are influenced by size and source of sampling, there is a need for a large-scale effort to aggregate appropriate and adequate samples by taking into account social hierarchy and endogamy prevalent in Indian subcontinent.

## Supplemental Information

10.7717/peerj.12294/supp-1 Supplemental Information 1 Details of the populations and number of samples of 1KGP and gnomAD included in the analysis of Very Important Pharmacogenes

10.7717/peerj.12294/supp-2 Supplemental Information 2 VCF file of exclusive SNPs belonging to VIP genes identified in GIH population through joint genotyping

10.7717/peerj.12294/supp-3 Supplemental Information 3 VCF file of exclusive SNPs belonging to VIP genes identified in ITU population through joint genotyping

10.7717/peerj.12294/supp-4 Supplemental Information 4 Population stratification of VIP genes at *k*= 3,5,6

10.7717/peerj.12294/supp-5 Supplemental Information 5 PCA clustering of genome-wide SNPs of 1KGP samples AFR in grey, EUR in blue, EAS in red, AMR1 in yellow, AMR2 in green, GIH in purple and ITU in black

10.7717/peerj.12294/supp-6 Supplemental Information 6 Complete list of SNPs belonging to Very Important Pharmacogenes (VIP) that show significant high allele frequency variation in GIH and ITU, when compared with other super-populations of 1000 genome project and gnomAD

10.7717/peerj.12294/supp-7 Supplemental Information 7 Complete list of SNPs belonging to Very Important Pharmacogenes (VIP) that show significantly low allele frequency variation in GIH and ITU, when compared with other super-populations of 1000 genome project and gnomAD

10.7717/peerj.12294/supp-8 Supplemental Information 8 Complete list of SNPs belonging to Very Important Pharmacogenes (VIP) ≥0.5 Fst in GIH/ITU populations with one or more super-populations of 1000 genome project and gnomAD

## Additional Information and Declarations

### Data Availability

The following information was supplied regarding data availability: We used publicly available data from 1000 Genomes Project and gnomAD databases: - The data is available at the IGSR FTP site found at https://www.internationalgenome.org/data following these keywords: data collections > 1000 genomes project > release > 20181203 biallelic SNV. - gnomAD: https://gnomad.broadinstitute.org/downloads#v3-variants
- PharmKB: https://www.pharmgkb.org/downloads.
