# Supplementary figures and images for: Genetic diversity of ‘Very Important Pharmacogenes’ in two South-Asian populations
Source: PeerJ. 2021 Nov 10;9:e12294. doi: 10.7717/peerj.12294 (PMC8590392; doi:10.7717/peerj.12294)

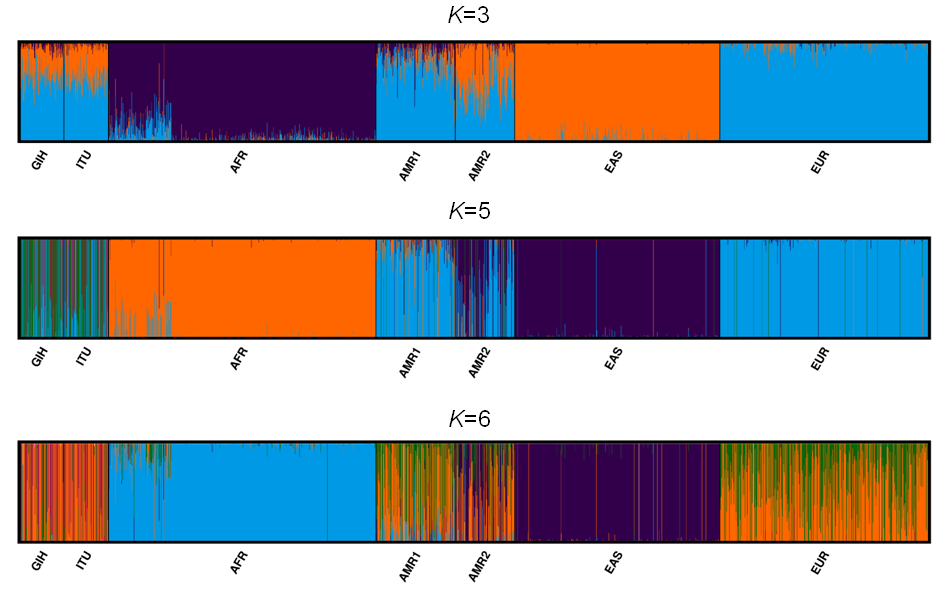

Supplement: Supplemental Information 4 [file peerj-09-12294-s004.png]

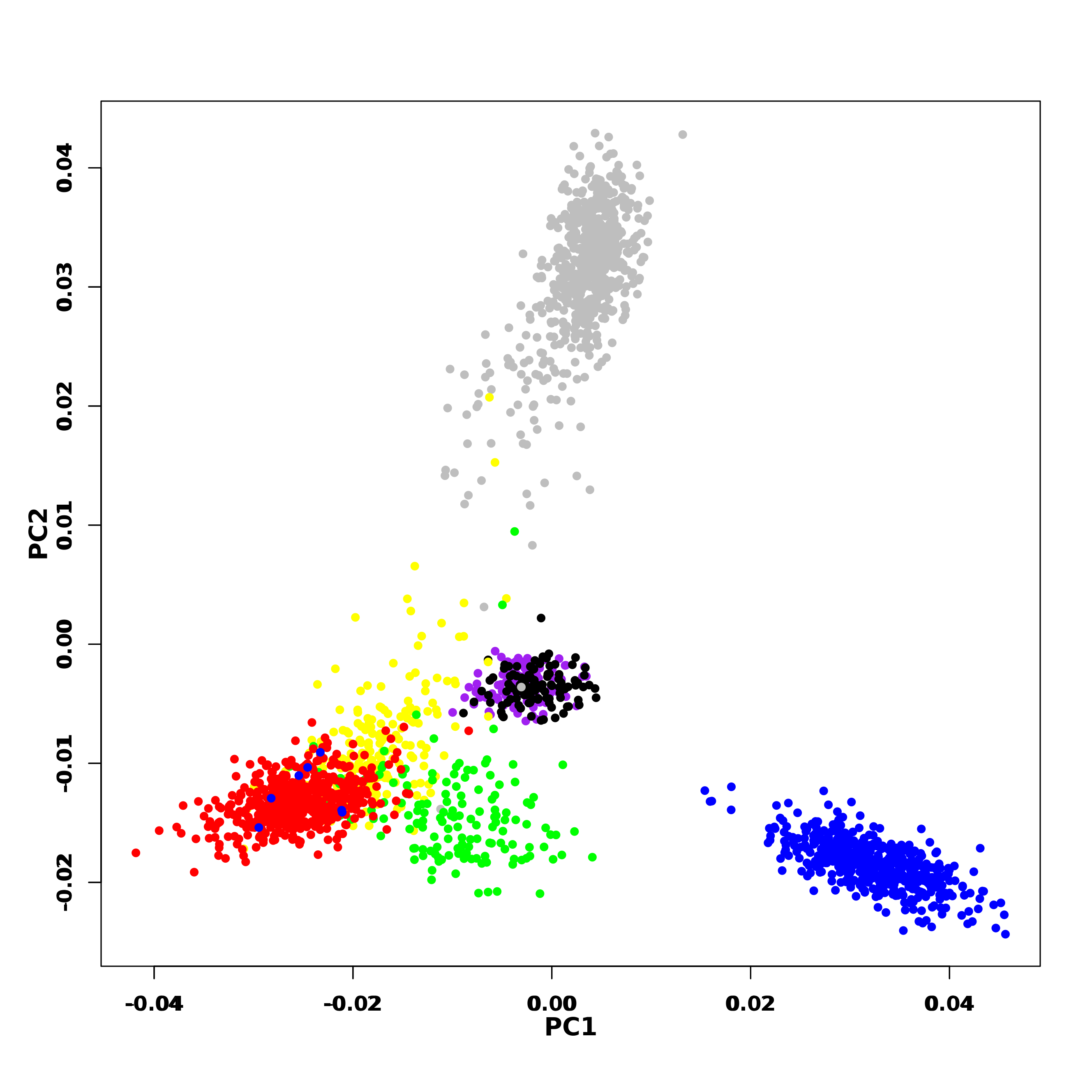

Supplement: Supplemental Information 5 — AFR in grey, EUR in blue, EAS in red, AMR1 in yellow, AMR2 in green, GIH in purple and ITU in black [file peerj-09-12294-s005.png]
